# Aflatoxin exposure in children living in Mirpur, Dhaka: data from MAL-ED companion study

**DOI:** 10.1038/s41370-018-0066-5

**Published:** 2018-09-05

**Authors:** Mustafa Mahfuz, Mohammed Ashraful Alam, Shah Mohammad Fahim, Md Amran Gazi, Mohammad Jyoti Raihan, Muttaquina Hossain, Patricia A. Egner, Pascal Obong Bessong, William A. Petri, John D. Groopman, Tahmeed Ahmed

**Affiliations:** 1 International Centre for Diarrhoeal Disease Research, Bangladesh (icddr,b), 68, Shaheed Tajuddin Ahmed Sarani, Mohakhali, Dhaka, 1212 Bangladesh; 2 Johns Hopkins Bloomberg School of Public Health, 615N. Wolfe Street, Baltimore, MD 21205 USA; 3 University of Venda, Thohoyandou, South Africa; 4 University of Virginia, Charlottesville, VA USA

**Keywords:** Aflatoxin, MAL-ED, Bangladesh, AFB1-lys, children

## Abstract

Dietary exposure to aflatoxin is implicated in growth faltering of children. Despite the high burden of childhood stunting in urban Bangladesh, there are no data on long-term exposure to aflatoxin. This study aimed to explore aflatoxin exposure levels in a group of children followed longitudinally. The current study used data and biospecimens collected during 2010–2014 as part of the MAL-ED birth cohort study in an urban slum of Mirpur, Dhaka where children were followed from birth to 36 months. AFB1-lysine adduct concentrations were determined by isotope dilution mass spectrometry from plasma samples collected at 7, 15, 24, and 36 months of age. The limit of detection was 0.5 pg of AFB1-lys/mg albumin. In 744 plasma samples, the geometric mean of AFB1-lysine/mg albumin was 1.07 pg (range 0.04–123.5 pg/mg albumin). The proportion of children with detectable aflatoxin exposure was 10.1, 20.9, 17.9, and 61.7% for 7, 15, 24, and 36 months, respectively. Reduction in breastfeeding prevalence (80% at 24 months vs. 38% in 36 months) corresponded with the high-level detection of AFB1-lysine at the age of 36 months. AFB1-lysine concentrations were the highest at the end of monsoon. This study reveals that 62% of children in slum settlement were exposed to aflatoxin by the end of the third year of life. High aflatoxin exposure was detected at the end of rainy season and with the introduction of family food. These findings suggest interventions to ameliorate the problem of chronic aflatoxin exposure including childhood stunting.

## Introduction

Linear growth faltering in children is a pervasive public health burden for low- and middle-income countries globally, and it is a consequence of a wide variety of factors such as undernutrition, poor hygiene, low socioeconomic status, local political instability, and repeated episodes of infectious diseases [1]. In addition to the widely recognized risk factors, dietary and environmental toxins may have a potential role in childhood stunting [2, 3]. Reports from Sub-Saharan Africa have implicated aflatoxin exposure with stunting among children [2, 4–6]. However, childhood stunting is highly prevalent in South and East Asia, as well as Sub-Saharan Africa where food-borne aflatoxin exposure is high [2]. Bangladesh has one of the highest prevalence rates of childhood stunting in the world. About 36% of children under the age of 5 years in Bangladesh are stunted or short for their age, while 15% are reported to be severely stunted [7].

Aflatoxins are the secondary fungal metabolites of *Aspergillus flavus*, *Aspergillus parasiticus*, and occasionally other *Aspergillus* species. Aflatoxin contamination has been well-documented in African staple foods such as sorghum, groundnuts, and maize, but has also been demonstrated in a variety of other food commodities including rice, wheat, pulses, sorghum, dried fruits, and spices in tropical and subtropical regions [8]. Hot and humid environmental conditions increase the likelihood of the growth of *Aspergillus* spp. and subsequent toxin production [2]. The extreme weather events in Bangladesh are believed to be conducive for the growth of *Aspergillus* spp. [9]. Studies have documented aflatoxin contamination of different food commodities of Bangladesh [10, 11].

Despite the fact that there is limited data on the level of aflatoxin exposure in Bangladeshi people, a recent study conducted in the north of Bangladesh observed a high level of detectable aflatoxin in children aged 2 years or less [12]. Childhood stunting is extremely high in a slum setting and a recent study observed that 50% of under-5-year-old children in slums are stunted [13]. Given the high burden of childhood stunting, there are no data on long-term exposure to aflatoxin as measured by aflatoxin B1-lysine (AFB1-lys) adduct in plasma samples from children in urban Bangladesh.

Based on metabolic transformation, different exposure biomarkers for aflatoxin B1 (AFB1) have been developed from blood, urine, and tissue samples [14]. The biomarkers commonly used are metabolites, DNA-adducts, or protein adducts [14, 15]. After ingestion, aflatoxin B1 is oxidized to highly reactive aflatoxin exo-epoxide by cytochrome P450 system in the liver. The epoxides then react with DNA to form guanine adducts or with serum albumin to generate lysine adducts [16]. DNA-adduct is a useful biomarker of AFB1 exposure but this method requires the availability of tissues to examine, which is a limitation [17]. The extent of the exposure of aflatoxin B1 can be measured, considering the dose-dependent manner of metabolites present in urine and blood [16]. Due to the short half-life, urine analysis can only be used in studies which detect acute exposure to AFB1 [17]. While AFB1-lysine adduct has a half-life of 20 days, it reflects chronic exposure [16]. Moreover, AFB1-lysine adduct occurs in a dose-dependent manner and accumulates to a steady level that correlates with AFB1 DNA-adducts in internal organs [17, 18]. Therefore, AFB1-lysine is the perfect less invasive biomarker of chronic aflatoxin exposure and has been used by almost all the studies conducted in the last decade.

This study was designed to explore the levels of aflatoxin exposure in a group of children followed longitudinally. This aflatoxin study took the advantage of a MAL-ED birth cohort study conducted in Bangladesh and used the framework of resources, data, and samples from MAL-ED study as it could control most of the variables associated with child growth and development [19]. “Etiology, Risk Factors and Interactions of Enteric Infections and Malnutrition and the Consequences for Child Health and Development” (MAL-ED) is a multisite project aiming to gain a better understanding of the risk factors for malnutrition, enteric diseases, and associated health consequences, including developmental impairment, in children of developing countries. MAL-ED is using a common research framework to test and develop hypotheses regarding enteric infections and, nutritional status and associations with childhood growth and development in eight developing countries including Bangladesh [20].

## Materials and methods

### Study design and participants

This MAL-ED aflatoxin study used data and biological samples from the MAL-ED birth cohort study in Bangladesh. In the MAL-ED birth cohort, the children were enrolled within 17 days of birth and followed longitudinally up to 36 months of age. Blood samples were collected at 7, 15, 24, and 36 months of age [20]. The study site is located in the Bauniabadh area of Mirpur, Dhaka. This area is densely populated, inhabited by poor and lower-middle-class families, and has suboptimal sanitary conditions with a high burden of childhood under nutrition and infectious diseases. The study area is located in a hot and humid tropical climate zone with three different seasons: hot pre-monsoon season (March to May), rainy season or monsoon (June to October), and winter season from November to February [19].

A total of 212 children from the MAL-ED cohort were available and 196 of them were followed through 36 months of age. Only children with available plasma samples who provided consent to use their samples for aflatoxin assays were enrolled in this study. The research protocol was approved by the Institutional Review Board of icddr,b and informed written consent was obtained from one of the parents of each participant. A complete description of MAL-ED study methodology including description of site, recruitment, inclusion and exclusion criteria, sampling method, and IRB approvals has been published elsewhere [19–21].

### Aflatoxin plasma biomarker assay

Aflatoxin B1-lysine adduct is a sensitive biomarker to determine long-term exposure to aflatoxin. All plasma samples were analyzed by an isotope dilution mass spectrometry method described in Groopman et al. [12]. In short, 200 µl of sample was mixed with an internal standard (10 µl × 0.1 ng of AFB1-D4-lys/ml) and pronase solution (Millipore Corp. Catalog #537088–100 µM) and incubated for 18 h at 37 °C. Samples were passed through a solid-phase extraction column (Waters Oasis^®^ MAX Cartridge 1 cc/30 mg, Catalog #186000366) and analyzed by UPLC with mass spectrometric detection system. The internal standard parent molecular ion ((M + H) + , m/z 461.3) fragments to yield a daughter ion at m/z 398.2. The AFB1-lys molecular ion (m/z 457.2) fragments to yield a daughter ion at m/z 394.1. Three quality control samples from rats dosed with AFB1 were run daily. The limit of detection was 0.5 pg of AFB1-lys/mg albumin.

### Surveillance and feeding data

In the MAL-ED cohort study, illness and infant feeding data were collected through twice weekly home visits. Detailed information on dietary and morbidity surveillance was published by Richard S et al. [21]. Infant feeding practice and information on the consumption of breast milk was collected during biweekly visits. A complete description of the methodology on infant and child feeding practice was described by Caulfield et al. [20]. From month 9 to 36, a monthly 24-h recall method was used to collect quantitative data on child feeding and nutrient intakes [22].

### Socioeconomic status

Based on preliminary research data, MAL-ED investigators had developed a simple composite index to measure the sum total of the socioeconomic status of families that can be compared across the countries and continents. This is known as the WAMI index. It includes water–sanitation–hygiene, maternal education status, monthly income, and asset status. Complete description of the WAMI index has been well described elsewhere [23].

### Statistics

In order to measure aflatoxin (AF), the blood sample was drawn when the sampled children were 7, 15, 24, and 36 months old. Descriptive statistics was used to present the characteristics of the sample. Bivariate analyses were conducted to observe the effect of different food groups and other confounders on AF concentration. Multivariate analysis in the form of generalized estimating equation (GEE) was used to understand the contribution of different food items to AF concentration after adjusting for potential confounders. As the blood sample was drawn and AF was measured at four time points, the GEE approach ensured the controlling of the correlation between the measurements taken at different time points. Only those variables, whose statistically significant bivariate association with AF was confirmed, were regressed in the multivariate model. The multivariate model only had those samples whose AF was detectable during all time points. Statistical significance was declared when p-value was less than 0.05 and all analyses were conducted in STATA 13.0 (STATACorp.).

## Results

**Fig. 1:**
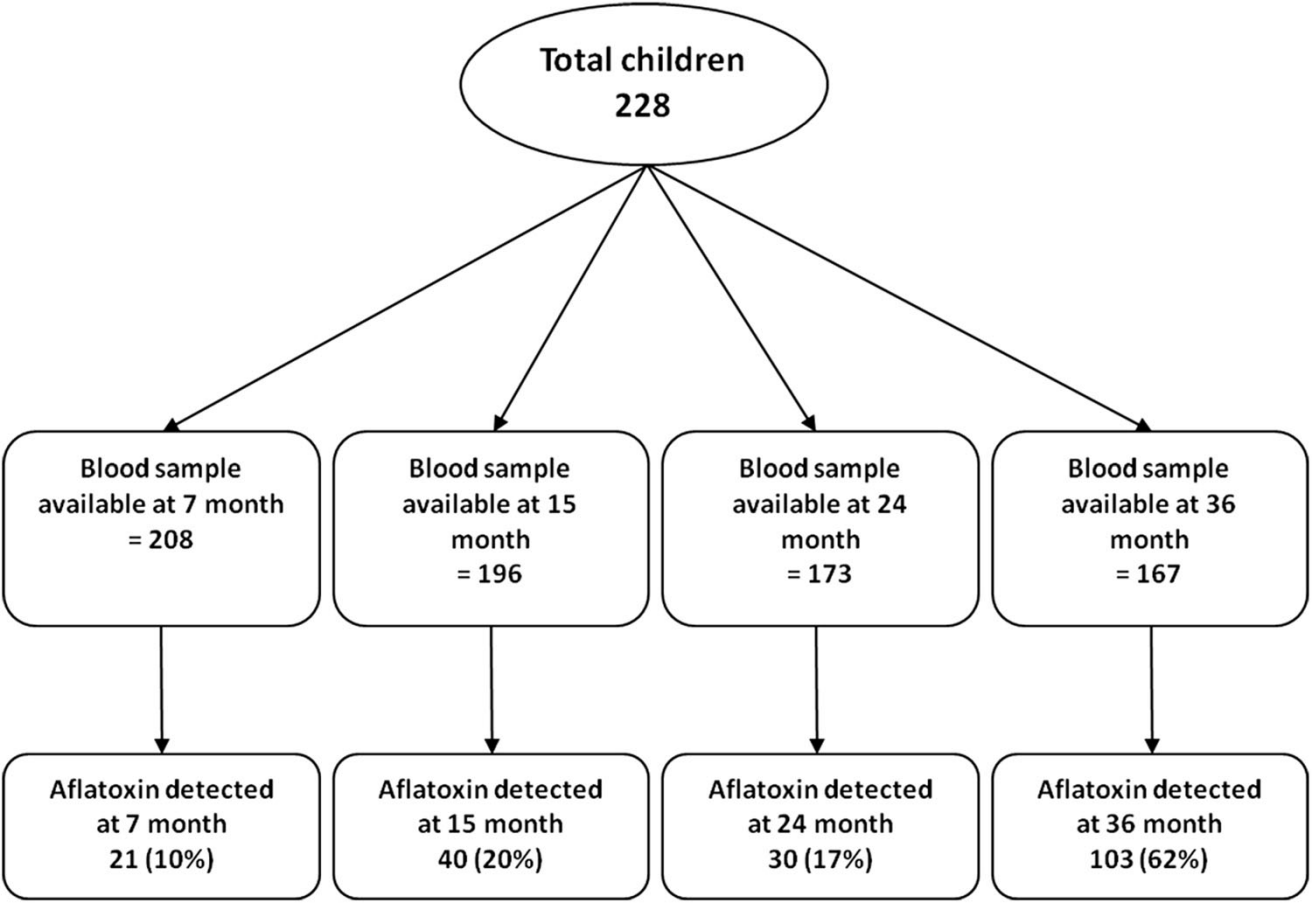
Study profile

In total, 228 children were enrolled in the study and 196 were followed up to 36 months of age. Blood samples were collected at 7, 15, 24, and 36 months of age. AFB1-lysine adduct (AFB1-lys) analysis was done from a total number of 744 plasma samples collected during 2010–2014 in four different time points (7 months, *n* = 208; 15 months, *n* = 196; 24 months, *n* = 173; 36 months, *n* = 167). The percentages of aflatoxin detection samples at those time points were 10, 20, 17, and 62%, respectively. Figure 1 shows the study profile.

**Fig. 2:**
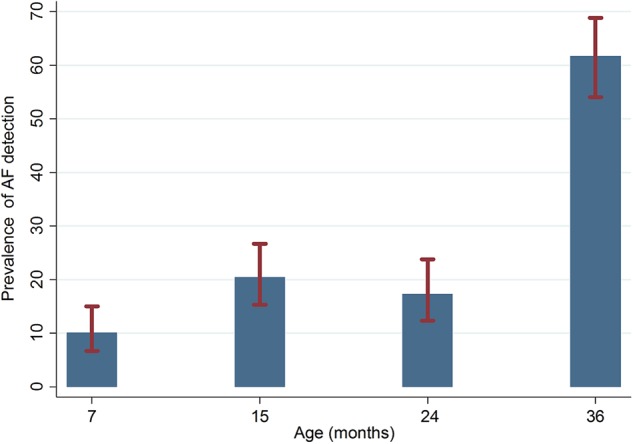
Prevalence of AF detection

The baseline descriptive characteristics of the study are detailed in Table 1. Nearly half of the study population was male and one in every five children had low birth weight. Around 16% of the children were stunted, 17% were wasted, and 21% were underweight at birth, indicating that the prevalence of undernutrition among the study samples was less than the national average. Table 2 and Fig. 2 show that the proportion of aflatoxin detection was similar in 15- and 24-months-old children. However, the lowest proportion of detection was for 7 months and the highest was for the 36-months-old children. In addition to data on the prevalence of AF in different age groups (Fig. 2), we also obtained the prevalence of breastfeeding in the same time period. The proportion of breastfeeding decreased with increasing age. At 7 months, the prevalence of breastfeeding was 97%, which decreased to 36% at 36 months.

**Table 1:** Baseline characteristics of children in the aflatoxin (AF) study in Mirpur, Dhaka, Bangladesh (*n* = 229)

Indicator	Value
Male sex, *n* (%)	108 (47.58)
Low birth weight, *n* (%)	50 (21.83)
Cesarean delivery, *n* (%)	47 (20.70)
Breastfeeding started within 24 h, *n* (%)	218 (95.20)
Weight for age *z* score < −2 at birth, *n* (%)	48 (20.96)
Length for age *z* score < −2 at birth, *n* (%)	37 (16.16)
Weight for length *z* score < −2 at birth, *n* (%)	37 (17.05)
Mother’s education < 5 years, *n* (%)	103 (44.98)
Monthly family income in USD, median (IQR)	101.26 (75.95, 126.58)
Number of food-insecure households, *n* (%)	57 (24.89)
Age at which the first solid food was given in days, median (IQR)	173 (154, 187)
Duration of EBF in days, median (IQR)	105 (58, 154)
WAMI index, mean ± SD	0.56 ± 0.12

WAMI index: Composite score for water–sanitation–hygiene, maternal education status, monthly income, and asset status. Complete description of the WAMI index has been given by Psaki et al. [18]

**Table 2:** Summary statistics of aflatoxin study

		Bangladesh (229/265)		
	Month-7	Month-15	Month-24	Month-36
Number of blood samples collected	212	197	177	168
Number of insufficient samples	4	1	4	1
Number of available serum samples	208	196	173	167
Below the level of aflatoxin detected	187	156	143	64
Aflatoxin detected	21 (10.10%)	40 (20.41%)	30 (17.34%)	103 (61.68%)
Summary of AF lysine (pg/mg)				
Mean	1.30	1.52	3.43	3.70
SD	1.50	1.47	11.83	12.99
Geometric mean	0.71	0.94	0.98	1.34
Median	0.83	1.14	0.95	1.17
IQR	(0.29–1.56)	(0.51–2.01)	(0.57–1.23)	(0.72–2.87)
Range	(0.09–5.79)	(0.06–6.35)	(0.15–65.60)	(0.09–126.54)

**Fig. 3:**
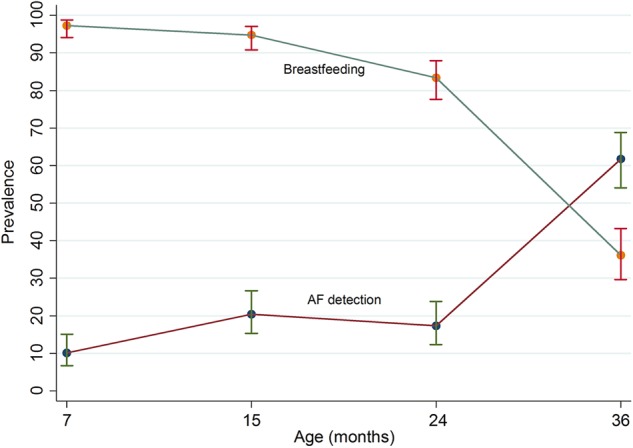
Prevalence of breastfeeding and AF detection

**Fig. 4:**
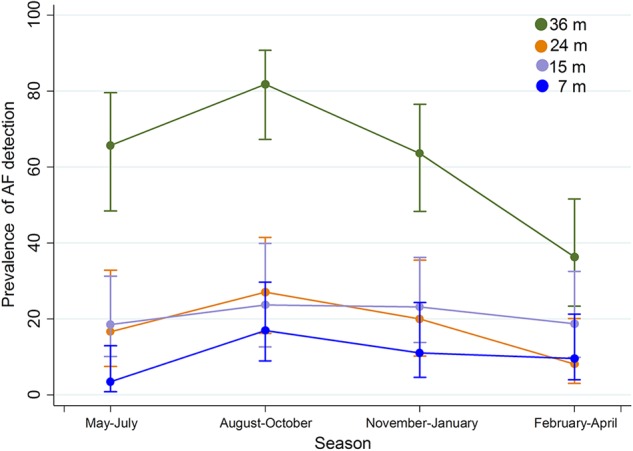
Seasonal variation in AF detection while children were 7, 15, 24, and 36 months old

Figure 3 shows the relationship between age, breastfeeding status, and AF detection status. The trend suggests an inverse relationship between breastfeeding status and AF detection. With increased age, the prevalence of breastfeeding decreases while the prevalence of AF detected increases. Moreover, between breastfed and non-breastfed infants across all four time points, aflatoxin detection was higher among non-breastfed infants (Table 3). Assessment of the seasonal influence on aflatoxin exposure is represented in Fig. 4. The prevalence of the highest AF detection was during August–October when any child turned 36 months old and the lowest was during May–July when children were 7 months old. In terms of seasonality of AF detection, the chi-square test dictates that there is no statistically significant difference between seasons for 7-, 15-, and 24-months-old children. However, there is a statistically significant difference (*p* < 0.001) in AF detection between seasons for the 36-months-old groups, with more detection in the months of August to October.

**Table 3:** Breastfeeding status and aflatoxin detection

Breastfeeding status	Aflatoxin detected^a^ (%, *n*)			
	7 months	15 months	24 months	36 months
Yes	9.14 (18)	19.44 (35)	16.43 (23)	67.24 (39)
No	50 (3)	50 (5)	81.25 (26)	58.72 (64)
*p*-value	0.016^b^	0.036^b^	0.752	0.281

^a^Based on the limit of detection of 0.5 pg of AFB1-lys/mg albumin

^b^Fisher’s exact test

Bivariate analysis showed that most of the food items had statistically significant association with AF concentration except for yellow vegetables and organ meats. Gender, WAMI index, and maternal education status were also not statistically significant (*p* > 0.05) in the bivariate analyses. However, in the multivariate model, only consumption of “sweets” was found to be significantly associated with AF [OR: 2.17 (95% CI: 1.27, 3.70), *p* < 0.05]. Both August–October [OR: 3.09 (95% CI: 1.83, 5.21) *p* < 0.05] and November–January [OR: 2.03 (95% CI: 1.16, 3.55), *p* < 0.05] were significantly associated with AF concentration. Breastfeeding status [OR: 0.35 (95% CI: 0.24, 0.52)] also had a significant protective effect against AF (Table 4).

**Table 4:** Bivariate and multivariate relationships between aflatoxin exposure levels and variables related to socio–economic status, dietary intakes, maternal education, and seasonality

	Unadjusted odds ratio (95% CI)	*p*-value	Adjusted odds ratio^e^ (95% CI)	*p*-value
Grain^a^	2.87 (1.02, 8.11)	0.046	0.53(0.15, 1.95)	0.341
Root	3.56 (2.03, 6.26)	0.000	0.91(0.34, 2.45)	0.854
Yellow vegetable	0.80 (0.50, 1.26)	0.328		
DGLV^b^	2.07 (1.49, 2.88)	0.000	1.13(0.76, 1.69)	0.534
Legume	3.41 (1.98, 5.87)	0.000	1.41(0.58, 3.44)	0.448
Yellow fruits	1.61 (1.15, 2.26)	0.006	0.71(0.46, 1.09)	0.119
Other fruits and vegetables	4.32 (2.45, 7.61)	0.000	1.71(0.80, 3.68)	0.166
Meat	2.01 (1.44, 2.80)	0.000	1.21(0.80, 1.83)	0.358
Organ meat	0.61 (0.23, 1.70)	0.354		
Egg	2.83 (1.97, 4.06)	0.000	1.58(0.97, 2.58)	0.065
Fish	2.49 (1.73, 3.58)	0.000	1.06(0.61, 1.82)	0.843
Dairy	2.60 (1.72, 3.92)	0.000	0.91(0.51, 1.63)	0.751
Sweet^c^	3.75 (2.41, 5.84)	0.000	2.17(1.27, 3.70)	0.004
Breastfeeding	0.26 (0.18, 0.38)	0.000	0.34(0.23, 0.50)	0.000
Gender, female	1.12 (0.82, 1.53)	0.472		
WAMI^d^ index	0.70 (0.18, 2.81)	0.619		
Season (Ref: February–April)				
May–July	1.33 (0.79, 2.24)	0.285	1.61(0.92, 2.83)	0.095
August–October	2.69 (1.66, 4.37)	<0.001	3.02(1.79, 5.11)	0.000
November–January	1.95 (1.17, 3.24)	0.010	2.04(1.17, 3.58)	0.013
Mother’s education (Ref: no education)				
Primary incomplete	1.03 (0.62, 1.73)	0.902		
Primary complete	1.13 (0.65, 1.99)	0.647		
Secondary incomplete	1.06 (0.63, 1.77)	0.838		
Secondary complete or higher	0.96 (0.42, 2.16)	0.916		

^a^Grains: rice, porridge, bread, noodles, or other foods/drinks made from grains

^b^DGLV: Dark green leafy vegetable

^c^Sweet: any sugary foods such as pastries, cakes, or biscuits

^d^WAMI index: Composite score for water–sanitation–hygiene, maternal education status, monthly income, and asset status. Complete description of the WAMI index has been given by Psaki et al. [18]. Generalized estimating equation (GEE) was used to understand the contribution of different food items to AF concentration after adjusting for potential confounders

^e^Only those variables, whose statistically significant bivariate association with AF was confirmed, were regressed in the multivariate model. The multivariate model only had those samples whose AF was detectable during all time points

## Discussion

Chronic exposure to AF may be implicated in growth impairment which was evident in studies conducted in sub-Saharan Africa [4, 5, 24, 25]. But data on community-based estimation of AF exposure and its implication on childhood growth are very limited in South Asia. A study conducted in semi-urban neighborhood of Nepal demonstrated chronic AF exposure among children [26]. Another study performed in rural areas of Nepal and Bangladesh illustrates ubiquitous exposure of AF in both the countries [12]. Such type of chronic exposure may result in malnutrition and growth faltering in children. Most of the works related to this was done in African countries and very little is known about the AF exposure in urban South Asia. Our study is the first to explore the chronic exposure of AF in an urban slum of Mirpur, Bangladesh. In the present study, the geometric mean of plasma AFB1-lysine concentration at 36 months of age (1.34 pg/mg) was lower than the geometric mean of 4.06 pg/mg, reported for the same-age children in Nepal. However, both the studies provide evidence of chronic aflatoxin exposure among children [26].

Previously, AFB1-alb biomarker by the ELISA method was commonly used for the detection of aflatoxin. But the advent of AFB1-lys albumin adducts biomarker made the detection of AF in dietary constituents more convenient. AFB1-lys pronase digestion product is typically measured by the LC–MS/MS method which is approximately 2.6 times more specific than AFB1-albumin measurement by ELISA. So, the different analytical methods that exist have varying sensitivities and specificity, which should be considered when making a comparison of AFB1-lys levels in different population-based studies worldwide. For instance, if we multiply the values for AFB1-lys in our study by 2.6, for comparative purposes with other aflatoxin-stunting studies that all use ELISA methods, then the geometric mean is equivalent to 9.62 with a range of 0.234–329.004 pg/mg albumin. Although the mean is lower than that previously reported in African populations, the range is similar to those observed in one study conducted in 785 infants born in Ghana.

This study provides evidence that children in an urban slum of Mirpur are chronically exposed to aflatoxin from 7 to 36 months of age. AF exposure was the lowest at 7 months and it tends to increase with increasing age. The prevalence of AF exposure was the highest (62%) at the end of the third year. At 7, 15, and 24 months of age, there were relatively lower percentages of detectable AF which is probably due to the major contribution of breast milk to the child’s diet at this age. We assessed that the prevalence of breastfeeding reduced almost threefold (2.7 times) from 7 to 36 months of age. In contrast, AF exposure increased over the same time period with an increased introduction of family diet. Both the bivariate and multivariate analysis showed that reduction in breastfeeding is likely to be associated with an increase in aflatoxin exposure in Mirpur. This is in accordance with the findings of previous studies conducted in Sub-Saharan Africa, which reflected that weaning onto family food increases AF exposure [4, 5, 24, 25]. AF exposure is found to be significantly associated with weaning status in African studies because of the increases in consumption of maize and peanut-based food during the weaning period. It is noteworthy that even the children who were on partial breastfeeding had an increased level of AF-alb, possibly reflecting the increasing proportion of total food consumption coming from the weaning and family foods as the child becomes older [24, 25].

Dietary contamination of AF was detected in a variety of foodstuffs in Bangladesh [10, 11]. In consistence with that, 62% of serum samples were tested positive for AFB1-lys in our selected cohort. Most of the regularly consumed food items in Bangladesh including grain, legumes, meat, egg, and fish were found to be significantly associated with AF concentration except for yellow vegetables and organ meats. This finding correlates the most likely source of aflatoxin exposure during the weaning period. In the multivariate model, only “sweets” was found to be significantly associated with AF. As per our dietary data collection questionnaire, “sweet” food means any sugary food or drink that is sweet such as pastries, cakes, or biscuits. However, the major staple crop in this region is rice, which is relatively less susceptible to aflatoxin contamination. It was found that grain (that includes rice and wheat) has a significant association with AF contamination in our study at the bivariate level. But in Nepal, no association was demonstrated with the exposure of AF in relation to weaning status. It was reported from the MAL-ED cohort that Nepalese children had the highest consumption of formula feeding and solids/semisolid foods in the first month of their life. This early initiation of artificial feeding can be an explanation of no association between AF exposure and weaning status. Another potential reason can be the type of foods that is consumed in Nepal during the weaning period. Rice is the primary grain in Nepal which contains a very low amount of AF [26].

Our study result depicted the seasonal variation of AF with a highest detection during August–October among the children of 36 months. November–January was also significantly associated with AF exposure. AF is a potent mycotoxin that can contaminate staple grains in hot and humid climate. Bangladesh belongs to a subtropical climate zone with warm temperature and high humidity during monsoon and post-monsoon seasons [9]. Heavy rainfall and wet environment during monsoon provides an optimal condition for fungal growth and AF contamination, which may reveal immediately after the rainy season. Additionally, post-harvest storage of food crops in hot and humid conditions promotes mycotoxin formation and AF contamination in tropical countries [24]. In Bangladesh, the rainy season lasts from June to September and post-monsoon season extends up to October and November [9]. The increase in AFB1-lys levels during this period indicates the seasonal availability of aflatoxin contamination in staple grains. A similar type of seasonal variations was also observed in Nepal and African countries [5]. However, seasonal variation was not found significant for the children at 7, 15, and 24 months old.

There have been some limitations of the described study. First, this work would be more conclusive with additional dietary intake information and quantitative determination of the aflatoxin in those food items for each individual child. In fact, to fully distinguish the effects of the toxin from other confounding factors in the diet would require a randomized intervention study where the impact of lowering aflatoxin exposure on child immunity, growth, and disease susceptibility can be assessed. This would also permit a better understanding of the relative contribution of aflatoxin to growth impairment in relation to other important determinants in these communities.

In conclusion, the results of this study confirm that 62% of children are chronically exposed to aflatoxin in the third year of life. This is the first to report on AF in children from urban Bangladesh assessed by a robust mass spectrometry-based albumin adduct biomarker. High aflatoxin exposure was detected at the end of the rainy season and during the post-monsoon period. Reduction of breastfeeding and starting of family food was positively correlated with aflatoxin exposure. These data strongly suggest further exploration of the sources and determination of the threshold value of aflatoxin contamination in the staple grains in Bangladesh. However, the findings of this study point to critical windows for interventions to ameliorate the problem of chronic aflatoxin exposure and evaluate its impact on childhood growth. Further analysis will look into the association of aflatoxin exposure in relation to growth of young children in the same population.

### Availability of data

Due to restriction in icddr,b’s data access policy in regard to participants identifying information, data are available upon request from the Research & Clinical Administration and Strategy (RCAS) of icddr,b (http://www.icddrb.org/component/content/article/10003-data-policies/1893-data-policies) for researchers who meet the criteria for access to confidential data.
